# Crisis Ahead? Why Human-Robot Interaction User Studies May Have Replicability Problems and Directions for Improvement

**DOI:** 10.3389/frobt.2022.838116

**Published:** 2022-03-11

**Authors:** Benedikt Leichtmann, Verena Nitsch, Martina Mara

**Affiliations:** ^1^ LIT Robopsychology Lab, Johannes Kepler University Linz, Linz, Austria; ^2^ Institute of Industrial Engineering and Ergonomics, RWTH Aachen University, Aachen, Germany

**Keywords:** metascience, replicability crisis, theoretical human-robot interaction, philosophy of science, open science, social robotics

## Abstract

There is a confidence crisis in many scientific disciplines, in particular disciplines researching human behavior, as many effects of original experiments have not been replicated successfully in large-scale replication studies. While human-robot interaction (HRI) is an interdisciplinary research field, the study of human behavior, cognition and emotion in HRI plays also a vital part. Are HRI user studies facing the same problems as other fields and if so, what can be done to overcome them? In this article, we first give a short overview of the replicability crisis in behavioral sciences and its causes. In a second step, we estimate the replicability of HRI user studies mainly 1) by structural comparison of HRI research processes and practices with those of other disciplines with replicability issues, 2) by systematically reviewing meta-analyses of HRI user studies to identify parameters that are known to affect replicability, and 3) by summarizing first replication studies in HRI as direct evidence. Our findings suggest that HRI user studies often exhibit the same problems that caused the replicability crisis in many behavioral sciences, such as small sample sizes, lack of theory, or missing information in reported data. In order to improve the stability of future HRI research, we propose some statistical, methodological and social reforms. This article aims to provide a basis for further discussion and a potential outline for improvements in the field.

## 1 Introduction

The year 2011 hit psychology hard as a row of events led to something that would later become known as the “replicability crisis” (Świątkowski and Dompnier, 2017; Romero, 2019; Wiggins and Christopherson, 2019). A significant portion of quantitative studies that tried to replicate findings of classic psychological experiments from prestigious journals had failed to find the effects that were reported in the original work. For example, in a multi-lab study the Open Science Collaboration (2015) concluded that only 39 of 100 effects had successfully replicated the original findings. Psychology is not alone with this worrying result—many other disciplines such as neuroscience (Button et al., 2013), economics (Ioannidis et al., 2017) or cancer research (Begley and Ellis, 2012) have also found problems of replicability. A Nature survey of 1,576 researchers from various disciplines (e.g., medicine, biology, and engineering) showed that 90% of researchers think that there is a crisis in science, 53% even think that this crisis is a significant one (Baker, 2016). The conclusion is clear: The crisis goes beyond replicability, it is a crisis of confidence and it is affecting many scientific disciplines primarily those that are based on the study of human behavior and rely heavily on quantitative methods (Ioannidis, 2005). While HRI is a very heterogeneous research field with many different disciplines and perspectives concerning, for example, design processes, hardware and software aspects, the study of the interaction between human users and the machine systems in user studies (and thus also a social and behavioral perspective) is also a significant part of the discipline (Sheridan, 2016; Bartneck et al., 2020). As other disciplines focusing on human behavior and the use of quantitative methods had such problems in replicability, this also raises the question of whether quantitative HRI user studies could be affected by replication problems.

Replicability is at the core of our understanding of science, and if our research is not replicable, we would have to conclude that 1) we do not really know as much as we hoped, that is our results are less generalizable than originally assumed, 2) funding might get wasted by building on research that is not reliable, and 3) public trust in science might be damaged, that includes trust of policy makers and funding agencies (see Wingen et al., 2020). Replication has thus the functions to control for sampling error, to control for artifacts, to control for fraud, to generalize results to different populations and situations, and to verify the underlying hypothesis (Schmidt, 2009). The replicability crisis is thus a serious topic that requires to break boundaries in form of discourse, reforms and action (Romero, 2019; Wiggins and Christopherson, 2019).

While the replicability crisis is intensely discussed in other disciplines, HRI as a research community is only starting a discussion of replicability and reforms when conducting user studies. Up to now, only a small number of articles has been dealing with this issue and many of them only cover a small fraction of the whole picture (Baxter et al., 2016; Irfan et al., 2018; Leichtmann and Nitsch, 2020a, 2020b; Stower et al., 2021; Ullman et al., 2021). For example, while scholars have pointed out problems and recommendations for laboratory experimentation (e.g., Eyssel, 2017) or statistical analysis (e.g., Hoffman and Zhao, 2021), other aspects have been neglected (e.g., philosophical-epistemological aspects), or have been discussed on an eclectic basis or briefly because an in-depth discussion of causes and reforms would have been beyond the scope of these articles (e.g., Leichtmann and Nitsch, 2020b). Additionally, besides some conference workshops (from recent years) and single brief mentions in other articles, work specifically dedicated to the problem of replicability has been rare in the context of HRI user studies (i.e., a search for “replicability” and “Human-Robot Interaction” in scientific databases only shows a limited number of results in 2021). Considering the serious consequences, the growing evidence, and reforms in other disciplines, HRI is also in need of more intense discussions on the reliability of our research results of HRI user studies, and we as HRI researchers need to consider learnings from this confidence crisis in our research programs to a higher degree. Such intense reflections and discussions on the replicability crisis have led other fields such as social psychology to improvements in methods, standards and practices (Motyl et al., 2017). To foster such a discussion also in HRI, this article’s goal is threefold: 1) A first goal is to inform other HRI researchers about the replicability crisis, its origin, its causes and its significance by bringing together a range of different reflections and insight on this topic from other disciplines in the behavioral sciences or philosophy to raise awareness and to establish a common understanding of problem. 2) Additionally, this article shows that the replicability crisis might be equally serious in HRI user research through an analysis of the recent HRI literature, and, 3) more importantly, we give an overview of solutions proposed by reformers in other disciplines that can also lead HRI to more stable research results. However, as HRI is a diverse and interdisciplinary discipline a process of change in HRI research methods already seems to be underway. Based on this, we outline possible further developments.

This is done in three steps. First, the article is giving an overview of the replicability crisis (or better “crisis of confidence”; Pashler and Wagenmakers, 2012; Wiggins and Christopherson, 2019) and its causes from a meta-scientific perspective. Second, we then estimate the replicability of HRI research (i.e., user studies) by comparing HRI research practices to those of other disciplines with replicability problems, by systematically analyzing meta-analyses in HRI from the past 2 years to detect patterns that indicate replicability problems, and by summarizing results from replication studies in HRI—turns out HRI, like many other disciplines, is facing problems of replicability and confidence and causes are manifold. Third, we will give an overview of suggestions for reforms to overcome the replicability crisis and to improve HRI user studies toward more stability (e.g., Romero, 2019; Wiggins and Christopherson, 2019). In summary, this article aims to provide a basis for further discussion and a potential outline for improvements in the field that need to be tackled for HRI research to become more reliable and consequently, for researchers to become more confident in their results. While replicability itself is an important value from many perspectives including for example the replicability of run-able code in software development, this article focuses on replicability aspects of HRI research focusing on user studies, and thus bringing the discussion and learnings specifically from the behavioral sciences into the field of HRI. As there has not been many articles specifically dedicated to the replicability of user studies in HRI and knowledge of these problems varies widely within the HRI community compared to other closely-related disciplines due to the youth of HRI as a discipline and due to the diversity of its community, this article can be a valuable contribution to a timely and relevant discussion for the HRI community.

## 2 What is the Replicability Crisis?

While there have been several crises in the history of science and there is a continuous discussion about methodological weaknesses (Greenwood, 1982; Eagly and Riger, 2014), a row of events led to a new crisis of confidence in psychology, often referred to as “replicability crisis”. This eventually forced psychology to act and resulted in various reforms (Pashler and Wagenmakers, 2012; Świątkowski and Dompnier, 2017; Romero, 2019; Wiggins and Christopherson, 2019). After a brief description of the term “replicability crisis” in the introductory section, this section goes into more detail on how this crisis emerged historically and what causes had been identified for this crisis in the behavioral sciences in order to understand the complexity of this crisis more fundamentally. This understanding is then used in the following sections for a reflection and analysis if HRI user studies are facing similar problems—as a first estimator of the status quo—and to derive possible solutions to improve research reliability.

### 2.1 Historical Roots of the Replicability Crisis: How Bad is it?

For psychology, the crisis took off in 2011: Deryl Bem published improbable empirical results on para-psychological phenomena in a prestigious journal (Bem, 2011) causing scholars to question currently common research practices (see Wagenmakers et al., 2011). Second, Simmons et al. (2011) published an article in which they demonstrate how it is possible to find significant results for any hypothesis by using so called questionable research practices (QRPs). QRPs is unjustified and undisclosed flexibility in data collection and analysis that allows to present any effect (even if absurd) as statistically significant. Third, cases of real fraud called into question the quality of control mechanisms in science and challenged trust in the system (Callaway, 2011; Wicherts, 2011). While these events gave some hints that a considerable proportion of scientific literature might be flawed, multisite projects such as the Many Labs Projects aimed to assess the replicability, and thus reliability, of research results systematically (Klein et al., 2014; Ebersole et al., 2016; Klein et al., 2018). Overall, many replication studies found a substantial decline in effect sizes compared to the original studies (Open Science Collaboration, 2015). This called into question the reliability and validity of research findings. Therefore, more effort is needed to sort out false-positives and to examine the stability of effects. To do so, it is needed to understand the reasons for the crisis—and this understanding will then also enable to identify if HRI user studies might be similarly affected.

### 2.2 Causes of the Replicability Crisis: A Mix of Factors

The replicability crisis is a complex phenomenon and is the result of a multitude of factors, pointing toward a lot of problems concerning fundamental epistemological assumptions, theory, measurement, statistical analyses, and reporting standards up to the publication processes and incentive system in science. Some authors suggest that the crisis goes beyond replication problems but affects the confidence in science in general, and thus the term “confidence crisis” would be more appropriate (Pashler and Wagenmakers, 2012; Wiggins and Christopherson, 2019).

#### 2.2.1 Science is a Social System

In order to understand the roots of the replicability crisis, it is important to note that science is not objective, but science needs to be understood as social system that is also influencing how we conduct, analyze and interpret our studies. Research is conducted by human researchers, which are part of a social world, and thus their work is also driven by values, goals, and theoretical assumptions. That means, what we consider as “knowledge” does not only stem from the phenomenon in question but is constructed by researchers and is thus shaped by their philosophy of science and the scientific social system. Many philosophers thus reject the assumptions of positivists that hold the “*general commitment to the rationality of scientific theory change and the idea that there is some kind of scientific method*” (Ladyman, 2019). In contrast to this, post world-war philosophers emphasized the social nature of science. Observations are thus not objective but “theory-laden” (Ladyman, 2019), that means that theory determines what is observed and how it is observed (Hanson, 1958; Ladyman, 2019). Summarizing work of philosophers Thomas Kuhn (1962) and Paul Feyerabend (1975), Ladyman (2019) thus concludes that “*evidence is somehow in the eye of the beholder*” and “*scientific theory choice owes at least as much to individual, social, and political values and idiosyncrasies as it does to the evidence*” (Ladyman, 2019). That does not mean that evidence is just a social product. Post world-war philosophers accept the idea of an external reality independent of a human observer. However, they emphasize that observations are biased and error-prone (Eagly and Riger, 2014).

#### 2.2.2 Publication Bias

One key issue that led to the replicability crisis, is directly rooted in the socio-historical nature of the scientific system—it is the way scientific results get published and rewarded that is also determining what will later be considered “knowledge” in the literature. The literature is thus socially shaped by the publication process (Meehl, 1990b; Eagly and Riger, 2014). This is not new. Paul E. Meehl pointed out that selective bias in submitting reports for publication or the selective editorial bias lead to a biased overall literature, distorted effects in the literature, wrong conclusions, and a biased basis for further work (Meehl, 1990b). These are two of ten factors that Meehl (1990b) lists as causes that make summaries of research—in his view—almost uninterpretable as “net epistemic effect” (Meehl, 1990b). Habits such as favoring positive statistical results compared to null-results or favoring certain theories that are “en vogue” compared to others inevitably lead to a distorted picture in the literature because the outcome of a study rather than the quality determines if it gets published—leaving studies of high quality but with “unfavorable” outcomes unnoticed in the “file drawer” (Świątkowski and Dompnier, 2017; Wiggins and Christopherson, 2019). As a consequence, results of reviews or meta-analyses are then overestimated (Meehl, 1990b) and if a study then tries to replicate such an overestimated average effect, chances are that they will find a smaller effect—if any.

#### 2.2.3 Questionable Research Practices

The issue of publication bias is closely connected to another issue—questionable research practices (often abbreviated QRPs) (Romero, 2019). Because publication bias favors some results above others, researchers have incentives to obtain results of a certain structure (e.g., positive results that is statistically significant ones). As already mentioned, Simmons et al. (2011) showed that it is possible to present any effect as statistically significant due to researchers’ flexibility in data collection and analysis (“researcher’s degrees of freedom”). QRPs are thus undisclosed methods that allow to alter statistical results in a favorable direction due to unjustified flexibility.

These QRPs include reporting only studies or single effects that obtained significant effects, or p-hacking, that is misuse of data analysis techniques until results reach a level of statistical significance (and then only reporting these effects). This can be achieved by excluding single cases, by only considering some sub-samples until results turn significant, by testing multiple times using multiple independent variables and a variety of dependent samples, as well as testing for multiple moderator variables (Simmons et al., 2011; Wicherts et al., 2016). It is conceivable that the same techniques would also allow an HRI researcher to portray any new design feature of a robot as “positive”, for example by multiple testing with multiple evaluation criteria. Without necessary correction procedures for multiple testing, such methods drastically increase the family-wise error rates (Holm, 1979), that is the probability of at least one Type-I error and is thus much higher than the often pre-defined α-level of 0.05. Consider a researcher collected data and performs five statistical tests with a local α-level of α_local_ = 0.05, then the global α-level for the system of hypotheses H = {H1, … , H5} would be much larger 1−(1−0.05)^5^ = 0.23. Thus, *p*-values need to be adjusted to control for this multiple testing, otherwise the probability of false-positive findings increases. Another QRP is “hypothesizing after the results are known” (HARKing), that is exploratory and unexpected results are presented as confirmatory (Kerr, 1998). HARKing thus also means performing statistical tests without theoretical legitimization until a test turns out significant and presenting this finding as a priori expectation.

QRPs like p-hacking or HARKing will lead to a “degenerative research line” in a Lakatosian sense (Lakatos, 1976), that is a research program will not be theoretically and empirically progressive. For example, let’s say the main effect of an independent variable did not turn out statistically significant. But instead of reporting this null result, the researcher performs additional interaction effects with many variables and then finds a significant effect only for men older than 52 living in Upper Austria for no theoretical reason. Reporting this effect as a “novel finding” would not lead to scientific progress due to a posteriori legitimization of a purely data-driven effect with high chance of being false-positive. Such a research process would not only be inefficient but also misleading.

It is important to note that QRPs are often used intermittently and are a result of confirmation bias. For Romero (2019), QRPs are thus even more troublesome because they are widespread, and researchers tend to justify them based on motivated reasoning (Romero, 2019) (e.g., “The effect turned out non-significant, but I wasn’t even sure about it from the beginning, I should not report it.”).

#### 2.2.4 Low-Powered Studies

One general problem that is closely related to the mindless use of QRPs is the misunderstanding or misuse of Null-Hypothesis Significance Testing (NHST), as the American Statistical Association emphasized in a statement on *p*-values (e.g., the confusion of statistical significance with the importance of a result) (Wasserstein and Lazar, 2016). This in turn is also the basis of another large problem causing replicability crisis: Low-powered studies (i.e., studies with too small sample sizes to detect an effect).

Statistical power is the probability of correctly rejecting the null hypothesis given that the alternative hypothesis is true (Lakens and Evers, 2014). High statistical power is thus crucial for studies to be informative and to add progress to a cumulative science. Underpowered studies have low probability of finding a true effect. Even if an underpowered study observes an effect, such studies have only small predictive power and are rather overestimated, which is also known as “winner’s curse” (Ioannidis, 2008; Button et al., 2013). Underpowered studies thus lead to a higher rate of false-positive results and an inflation of effect sizes (Button et al., 2013), making underpowered studies a major driver in the replicability crisis.

It had been widely recognized that psychological studies had been underpowered (Maxwell, 2004; Świątkowski and Dompnier, 2017). The prevalence of underpowered studies in psychology is due to an overall rather small (true) effect size (Schäfer and Schwarz, 2019), and at the same time small sample sizes (Maxwell, 2004; Button et al., 2013). A meta-scientific study in social psychology, in which effects from 100 years had been summarized concluded that the average effect size in social-psychological studies is only *r* = 0.21 (Richard et al., 2003). Thus, a large sample size is necessary to reliably measure such an effect. For example, Schönbrodt and Perugini (2013) show that with small sample sizes, the trajectories of effect size estimates fluctuate widely. They thus recommend sample sizes of approximately 250 for correlations (Schönbrodt and Perugini, 2013). This means, if no prior information is available, researchers should rather expect small effects in their power analysis and aim for large sample sizes. Small effect sizes lead to even higher rates of underpowered studies, i.e., Button et al. (2013) estimated the average statistical power of neuroscientific studies to be less than 31%. Similarly, a significant amount of HRI user studies relies on empirical methods using NHST, too, so it is equally important that HRI studies are based on satisfactorily powered designs to be reliable and statistical power of HRI user studies needs to be examined.

#### 2.2.5 Lack of Theory

Thus far, the discussion of the replicability crisis and its causes mainly focused on methodological and statistical problems in the literature (Wiggins and Christopherson, 2019). As Scheel et al. (2021b) summarized “*Hypotheses were tested, but the tests were weak and their interpretations were warped, resulting in overconfidence and false inferences.*” (Scheel et al., 2021b). According to them, this revealed a much deeper and more fundamental problem: A lack of theory. For Muthukrishna and Henrich (2019) many studies in behavioral sciences seem to be based on guesswork, arbitrary heuristics, custom and folk intuitions. However, without a theory [a set of concepts and statements describing how phenomena relate to each other (Davis et al., 2015), or in other words, a set of concepts that are connected by functional or compositional laws (Meehl, 1990a)], a systematic cumulative science is not possible because there is no framework that allows researchers to narrow down the number of potential variables to the most central ones and to specify predictions detailed enough.

That means that studies are rarely specified enough in order to eliminate flexibility in data analysis, even if the hypotheses had been formulated a priori, and there is no knowledge about the strength of manipulations and variances of measures leading to arbitrary heuristics (e.g., expecting medium effect sizes by default) (Scheel et al., 2021b). This vagueness in the formulation of hypotheses due to the lack of theory might also be one reason why QRPs are often used even unknowingly. There is always some room of flexibility left and thus we talk ourselves into using QRPs.

Additionally, such a lack in theory might also be the reason why scientists have difficulties in agreeing whether a replication had been successful (Scheel et al., 2021b). The failure of a single study to replicate an effect does not necessarily mean that the effect does not exist. It is not possible to replicate a study under completely same conditions, as there will always be differences in the sample, time, location or manipulations. Thus, replications vary in a certain degree of “sameness” (Romero, 2019; Wiggins and Christopherson, 2019). Because of these differences it is possible that there exist “hidden moderators” that cause differences in outcomes. Without theory to systematically point to important variables, such moderators could remain unnoticed. After a failure to replicate findings, one could come up with an almost countless number of variables that could have moderated the difference. This happens especially if the underlying theories and their boundary conditions (that is the regions of the parameter space in which the theory applies), as well as their auxiliary premises are not specified clearly enough (Meehl, 1990b; Scheel et al., 2021b). It is unclear which factors are important for the prediction of an effect under which conditions. One can always come up with alternative explanations for (not) finding an effect, which can easily lead to a degenerative research line making a hypothesis *de facto* unfalsifiable.

It should be noted that the differences in contexts also include time. That means that effects might change due to social changes over time, and this is especially true for social effects (van Bavel et al., 2016). Effects that hold now might not hold in 10 years. For example, new factors might get important to predict user acceptance of robots with robotic technology getting improved and more commonly implemented over time that we might not be aware of now. In an extreme view, such as Gergen’s (1973), psychology might be a historical discipline rather than science, as for Gergen (1973) such knowledge cannot accumulate because it cannot transcendent its socio-historical boundaries, making generalizations impossible. This idea might be extreme; however, it shows the difficulty to estimate such influences of changes, control them in studies, and account for them in theories. Contextual differences are thus a driving force that affects some effects more than others. Especially for contextual sensitive effects (and there are many in HRI) a thorough understanding of the processes and the boundaries are needed for successful replication (van Bavel et al., 2016).

In sum, the problem of the replicability crisis is not only caused by methodological or statistical problems, but starts with a lack of theory: Concepts are not specified, relationships between concepts are unknown, and boundary conditions and auxiliary assumptions are unexplored or unnoticed (Scheel et al., 2021b). Scheel et al. (2021b) conclude that psychology might simply not yet be ready to test hypotheses but needs to work on these theoretical elements first. This will strengthen the “derivation chain”—in Meehl’s words—a conjunction of theoretical and auxiliary premises necessary to predict observable outcomes (Meehl, 1990b).

#### 2.2.6 Lack of Validation

Another necessary step before testing hypotheses is measurement. However, measurement in psychology and related disciplines is not easy, as studies mostly deal with latent variables, that is variables that cannot be directly observed (e.g., attitudes or affective states). Methods to measure latent constructs only reflect the construct in a probabilistic way and thus vary in accuracy. For the measurement of a construct to be valid, it needs a solid theoretical grounding and validity needs to be proven in a validation process (Cronbach and Meehl, 1955; Borsboom et al., 2004; Borsboom, 2008). In the worst case, when a measurement lacks such a validation process, chances are that it reflects the construct in question rather poorly but reflects one or more other constructs or just random noise. In such a case, effects fluctuate as a function of noisy measures, and will not replicate. Meta-studies have analyzed the social-psychological literature and found that measures did not always undergo a thorough validation process (Flake et al., 2017; Hussey and Hughes, 2020). Consequently, measures could be invalid (“hidden invalidity”), reflect noise or different constructs than expected, and thus the failure to replicate a finding could potentially be attributed to a variance in measurement noise. In HRI user studies often new scales need to be developed that are specifically tailored to HRI contexts (i.e., measuring attitudes toward robots), thus a solid validation process is also key for results of HRI user studies to be valid—if this is missing measurement scales could be invalid and thus causing replicability problems also in HRI.

#### 2.2.7 Problems in Reporting Standards

A further problem that needs to be mentioned concerns the communication of research (results). Besides the norms of scientific investigation, there is also norms of scientific reporting that have been violated and thus cause replicability problems. Such norms are, for example, the norm of descriptive completeness, the norm of accuracy and clarity (Hensel, 2020). For replication, communication is not necessary if the same authors conduct the replication study, but is crucial for replications by other research teams (Hensel, 2020). If the description of an experimental setup, the sampling procedure, exclusion criteria, procedures of data preparation and other details are missing, it is difficult for other teams of scientists to fully understand the study design and thus it is difficult to replicate it as closely as possible. Changes in details may already result in changes in effects that can explain a failure of replication.

This norm of completeness does not only mean the complete description of study plan and description of its conductance, but also includes the full description of the analysis and statistical results. This includes to disclose all analyses and results, both significant and non-significant ones, to disclose which analyses are based on a priori hypotheses and thus confirmatory, and which results are data-driven and thus exploratory (Maxwell, 2004; Simmons et al., 2011; Button et al., 2013), as well as completely reporting statistics including degrees of freedom and test-statistics, but also effect sizes and confidence intervals, not just *p*-values (Maxwell, 2004; Lakens, 2013; Lakens and Evers, 2014). However, meta-scientific studies show that statistical information is often misreported or contains errors, important parameters are omitted, and scientists refuse to share data (Hensel, 2020). Besides problems in reporting statistical details, the literature also reveals failure to provide complete research descriptions (Hensel, 2020).

Transparency does not only allow for better replications, but also enables other researchers (the readers of the publication) to evaluate the results and draw their own conclusions on the validity. Additionally, transparency is crucial for calculating meta-analyses or for theoretical integration in reviews. Besides problems in scientific investigation itself, problems in communication of research are thus another core issue in the confidence crisis (Wiggins and Christopherson, 2019; Hensel, 2020).

## 3 Confidence in HRI Research

Thus far, an overview of the replicability crisis in the behavioral sciences was given and causal factors had been described. It turns out, the crisis is worrying, and causes are manifold, including publication bias, QPRs, misunderstandings of NHST, low statistical power, lack of theory, lack of validation, as well as violations of norms of reporting.

In a next step, it will be shown that these problems also affect HRI research, that is HRI user studies involving the study of human behavior in human-robot contexts with quantitative methods. Thus, we as HRI researchers need to take action to tackle these problems, too, when conducting user studies or when researching human behavior, cognition or affect in HRI contexts. According to Hensel (2020), indirect and direct methods of assessment can be used to estimate the replicability and confidence in research results. Direct methods involve conducting replications, whereas indirect methods try to identify various parameters and their values that are known to affect replicability.

### 3.1 Same Methods, Same Problems?

A first indirect indication that HRI user studies may also face replicability issues lies in the analysis of the research methods used in HRI, its practices as well as the analysis of the incentive and publication system of the scientific community.

Although there might be some differences, it is assumed that the same incentives and publication processes exist in the HRI community as in most other empirical disciplines. The publication process favors novelty and significance, and the scientific system incentivizes the number of publications causing publication bias. Since the use of QRPs is favored in such a system, it is to be expected that the HRI community is not immune to QRPs either.

Additionally, many problems of the crisis in confidence in the behavioral sciences stem from quantitative laboratory experiments and NHST. While HRI is a diverse research field with many different research approaches, quantitative laboratory experiments are among the most used research methods in HRI (Baxter et al., 2016), including the use of human participants, the study of their behavior, and the use of NHST. That means, a large portion of HRI research deals with the same complexity of human behavior and it is conceivable that HRI is similarly lacking theory in describing and predicting behavior. The use of NHST in HRI research raises the suspicion that HRI research also suffers from methodological and statistical problems arising from such a misunderstanding and misuse of NHST.

Meta-scientific analyses showed that social psychology had bigger problems in replicating effects compared to other subfields such as cognitive psychology (Open Science Collaboration, 2015; van Bavel et al., 2016). Van Bavel and others (2016) argue that this is due to the context sensitivity of social effects. Additionally, the average effect sizes of social phenomena are rather low (Richard et al., 2003), making it even more difficult to replicate. Similarly, HRI research also heavily relies on social effects (Irfan et al., 2018), especially social robotics as a social robot interacts and communicates with humans following social norms (Bartneck and Forlizzi, 2004). Therefore, HRI research is also context sensitive and average true effect sizes in user studies are likely to be small, which leads to the conclusion that HRI research might have similar difficulties of replicability.

While this is speculative, results from meta-studies and direct estimations of replicability in replication studies draw clearer indications of the scope of the crisis.

### 3.2 Meta-Analyses in HRI Show Many Issues

An evidence-based indirect approach to estimate the reproducibility of a field is the analysis of meta-analyses (Hensel, 2020). In meta-analyses, the literature is systematically screened for a particular question or effect, and statistical methods are used to calculate certain parameters to estimate the stability of effects across studies. Through this systematic collection of studies from the literature, meta-analyses can also paint a representative picture of what topics and practices are currently custom. This makes it possible to assess which practices are frequently used in a discipline and thus to reflect whether they are the same practices that foster replication problems, such as flexibility in analyses (Hensel, 2020).

To identify parameters that are known to affect replicability and confidence in results (e.g., sample size or QRPs) and the values of those parameters (e.g., how large are sample sizes?) in the empirical HRI literature on user studies, meta-analyses are summarized and discussed. We used the abstract and citation database Scopus to systematically scan the literature from the past 2 years for meta-analyses using the search logic “meta-analysis” AND “human-robot”. Only meta-analyses were used that 1) analyzed user studies, 2) are based on studies with one or more robots, and 3) use systematic search strategies (i.e., defined search terms, databases, and inclusion/exclusion criteria). Taxonomies have been excluded from this analysis. In total, eight meta-analyses had been used to identify patterns of concern in the literature: A meta-analysis by 1) Leichtmann and Nitsch (2020a) on personal space in HRI with a total of *k* = 27 studies including *N* = 1,299 participants, by 2) Stower et al. (2021) on trust in child-robot interaction including *k* = 20 studies with *N* = 977, by 3) Ötting et al. (2020) on the effects of design features on HRI at work using *k* = 81 studies with *N* = 2,245, by 4) Esterwood et al. (2021) on personality effects in HRI with *k* = 26 studies and *N* = 1,611, by 5) Roesler et al. (2021) on anthropomorphism in HRI with *k* = 78 studies and *N* = 5,973, by 6) Hancock et al. (2021) on trust in HRI using *k* = 45 studies, by 7) Yuan et al. (2021) on the effect of robot-assisted cognitive training including *k* = 53 studies with approximately *N* = 1,166 participants, and by 8) Mou et al. (2020) with k = 40 studies on “robot personality”. We additionally analyzed a recent meta-analysis not yet on Scopus, but in press. Mara et al. (2021) explored the uncanny valley hypothesis in HRI based on *k* = 49 studies with *N* = 3,556 participants. In sum, the meta-analyses cover more than 100 studies with a range of topics in HRI (e.g., proxemics, trust, or anthropomorphism) published in a wide range of conferences (e.g., ACM/IEEE International Conference on Human-Robot Interaction, International Conference on Social Robotics, or International Symposium on Robot and Human Interactive Communication) or journals (e.g., International Journal of Social Robotics). This range of different meta-analyses allows to estimate the replicability of the field broadly by identifying problematic patterns. Although not all meta-analyses evaluated the quality of the studies systematically, all of them report several weaknesses in the literature that appear to be common.

Problems of HRI research mentioned in the meta-analyses are manifold. One of the most obvious problems concern issues of scientific reporting, that is original studies are not always transparent enough, fail to describe all important information, and violate the norm of completeness. Most meta-analyses mentioned that statistical details had been missing such as effect sizes, confidence intervals, or descriptive statistics (i.e., means and variances) (Leichtmann and Nitsch, 2020a; Ötting et al., 2020; Mara et al., 2021; Roesler et al., 2021; Stower et al., 2021), or were inconsistent (Hancock et al., 2021) to such an extent that some studies had to be excluded for meta-analytical calculations. Lack of information is not only crucial to integrate knowledge in meta-analyses. As has been discussed before, if important information is missing, studies are also difficult (or impossible) to replicate and make it hard to evaluate the reliability of results or to contextualize it.

Another major problem of HRI studies is the widespread lack of theory. Leichtmann and Nitsch (2020a) summarize that “*studies mostly lacked theoretically well-grounded considerations*” and that the selection of factors often seemed eclectic. Ötting et al. (2020) found the same problems in their meta-analysis as empirical studies failed to explain why the factors had an effect on human-robot interaction parameters. This lack of theory even starts with problems in conceptualizing and defining constructs (Leichtmann and Nitsch, 2020a; Mou et al., 2020; Stower et al., 2021). For example, Mou et al. (2020) criticized that personality had not been conceptualized and defined in many of the identified articles on personality in robots, some even used personality models that are outdated or have been criticized for their validity problems. In other examples, Leichtmann and Nitsch (2020a) found problems with the conceptualization of “personal space” in the proxemics literature and Stower et al. (2021) criticized inconsistencies and imprecision in defining constructs such as trust in their meta-analysis. Due to this imprecision, it can happen that what is called a construct just reflects random noise.

Another hint toward this lack of theory is the heterogeneity of effects. Many meta-analyses found that effects varied widely, that is high confidence intervals reflecting low precision around effect size estimates (Leichtmann and Nitsch, 2020a; Ötting et al., 2020; Stower et al., 2021). This variance in effects could point toward hidden moderators. However, without theory it is difficult to determine which factors are important moderators that need to be controlled or further explored. Without defining the boundary conditions, it is difficult to predict under which conditions an effect holds. This heterogeneity of effects can also be a hint that HRI research is strongly context-sensitive (as has been predicted due to its similarities to social psychology) which likely means that HRI user studies are hard to replicate, effects are not as stable as hoped or that results can hardly be generalized across contexts. Furthermore, user studies in HRI seem to rely on very specific study participants such as samples from WEIRD (Western, Educated, Industrialized, Rich, Democratic) populations (Esterwood et al., 2021; Stower et al., 2021) or student samples (Baxter et al., 2016; Mara et al., 2021) and might not replicate in other populations.

However, this heterogeneity of effects can also be a result of varying validity of measurement instruments. Similar to other behavioral sciences, many psychological constructs are measured in HRI user studies that cannot be observed directly. Such constructs are then often measured using self-report. It is assumed that many scales used in HRI did not undergo a thorough validation process. For example, Ötting et al. (2020) report that “*some studies use self-developed measures without reporting information on validity or reliability*”. Another problem is QRPs. Leichtmann and Nitsch (2020a) found in their meta-analysis that many tests with several predictor variables had been performed or that in some data analyses researchers controlled for variables without theoretical legitimization or adjustments. Additionally, it was sometimes unclear if all statistical results had been disclosed (Leichtmann and Nitsch, 2020a).

Finally, varying quality of studies also means variance in statistical power. As noted before, statistical power is a function of sample size, effect size, and alpha level (Lakens and Evers, 2014). Low power is thus most often the result of low effect size and low sample size while the threshold for statistical significance is usually set to 0.05 in NHST (Benjamin et al., 2018). Almost all meta-analyses (except those which did not assess sample sizes) revealed that sample sizes in HRI studies are oftentimes very small—too small to detect small to moderate effects reliably. For example, Stower et al. (2021) found a mean sample size of *M* = 46.52 (*SD* = 37.34, *Mdn* = 29), Leichtmann and Nitsch (2020a) found a mean sample size of *M* = 48.11 (*SD* = 34.18, *Mdn* = 37), and studies in Ötting et al.'s (2020) analysis have a mean sample size of only *M* = 27.72 (*SD* = 21.36, *Mdn* = 22). In line with this observation, Mara et al. (2021) report also a median sample size of just *Mdn* = 21 and about 75% of user studies on cognitive training reported by Yuan et al. (2021) had sample sizes lower than 20. At the same time, the meta-analyses reported here show that effect sizes in HRI user studies seem to be rather small to moderate in most cases (Leichtmann and Nitsch, 2020a; Esterwood et al., 2021; Hancock et al., 2021; Roesler et al., 2021; Stower et al., 2021). Another hint for expecting rather low effect sizes is that a large portion of HRI user studies examined factors including anthropomorphism, attitudes, personality, gender, and others—topics that have been studied in context of the social sciences and are known to have rather small effect sizes (Richard et al., 2003). Based on the findings of this analysis that 1) sample sizes are rather low in quantitative HRI user studies, especially for widely used correlational and between-subjects designs, and 2) true effect sizes are expected to be rather small, it can be inferred that many user studies in HRI are based on underpowered study designs. This was in part also a direct conclusion from some of the meta-analyses themselves reporting that, for example, studies on personal space in HRI or trust in child-robot interaction had been based on underpowered study designs (Leichtmann and Nitsch, 2020a; Stower et al., 2021). Therefore, a considerable proportion of HRI studies likely has only low predictive power, reports overestimated effect sizes, and has high probability of finding false-positive results. Another hint for a biased literature was also found in a funnel plot analysis by Esterwood et al. (2021).

To sum up, meta-analyses on HRI research report almost all the problems that cause low replicability and are determinants for a crisis in confidence. These meta-analyses reported that a subset of quantitative HRI user studies 1) violated norms of scientific reporting, 2) lacked theory including a lack of precise concept definitions, 3) used unvalidated measurement instruments, 4) seem to have used QRPs, 5) or were underpowered. These are all causes of the replicability crisis in other research fields. Of course, all these problems had not been found in every study (and even if problems are found studies may only show two or three of these problems, not necessarily all), but the assessment of such problematic patterns (those that cause replicability problems and problems of confidence) discussed in meta-analyses may indicate that a certain number of HRI user studies might also face replicability problems and problems of confidence in research results. Note that this is an inference/estimation based on the analysis of a sample of meta-analyses discussed above, and as for every analysis there are limitations. A limitation here is that only an indirect approach has been used by reviewing results of meta-analyses from certain areas in HRI. Therefore, this work can only be a first preliminary estimation of problematic patterns in HRI user studies. Similar to research with participants, a sample of studies had been drawn from the “population” of HRI user studies to derive first estimates, but—of course—the goodness of this estimation can vary. Further in-depth analyses on different aspects and from different metascientific perspectives are urgently needed—this is HRI needs more metascientific analyes and critical reflections similar to other disciplines that engage in reflective discussions about their own practices (e.g., Wagenmakers et al., 2011).

### 3.3 First Replication Studies in HRI Indicate Replicability Problems

While the indirect assessment of replicability through the analysis of meta-analyses “*have much more scope and provide a good approximation of the upper bound on estimates of replicability*” (Hensel, 2020), they do not assess replicability directly. However, replication studies are still rather rare in HRI to this day, especially larger multisite projects systematically testing the replicability of HRI studies across cultures and settings like the Many Labs projects in psychology. As there exist only a few studies, they do not allow for a solid estimation of the replicability of HRI studies, but some examples can be used to sketch some first trends and to elaborate the problem.

In a conceptual replication, Leichtmann and Nitsch (2020b) aimed to replicate the effect of social desirability in human-computer interaction. Instead of using a computer like in the original study, a humanoid robot was used. Although having a larger sample size of *N* = 107 based on a power analysis and stronger social cues (robot instead of computer), they did not find a significant social desirability effect. The replication study did thus not replicate the effects of the original work.

In a large replication project, Ullman et al. (2021) tried to replicate one of their own studies because they had doubts about the results due to the small sample size (*N* = 42) and low power of their original study. Therefore, they conducted three replications with larger sample sizes based on power-analyses to test the robustness of their findings: They conducted a conceptual replication using a different robot with *N* = 140, a direct replication using the same robot with *N* = 200, and an online comparison study with *N* = 396. All three studies failed to replicate most of the results of the original study. The authors conclude that the primary finding of their original study must have been a false-positive result (Type I error).

Another replication study was presented by Strait et al. (2020). Three different research teams conducted conceptual replication studies on the joint Simon effect with a robot in three different countries including Germany, United States and Mexico. Based on an a priori power analysis study samples with *N*_*1*_ = 47, *N*_*2*_ = 51 and *N*_*3*_ = 72 participants had been used. While one hypothesis on the joint Simon effect from psychology had been shown to be replicable, another hypothesis specifically on user perceptions of the robot had not been able to be explored in all three studies due to data loss in two of them. While this limits the estimation of replicability in this case, the studies allow conclusions to be drawn regarding the conductance of replication studies in HRI. To ensure replicability, the researchers documented their process in close detail by sharing documents with information on hardware requirement, physical study setup, data analysis code and more in order to reproduce the experimental setup. In doing so and additionally applying other measures such as a priori power analysis the researchers show how it might be possible to improve replication rates.

Both, Ullman et al. (2021) and Strait et al. (2020) emphasized that replications in the field of HRI are particularly difficult because of the large variety of robots that exist, their limited availability, the high costs, and the difficulties in providing run-able source code. Wijnen et al. (2020) therefore had a different attempt to replicate another HRI study. Instead of using the same social robot as the original work, they conducted a conceptual replication using a virtual reality version of the robot. Again, the replication study did not find the same results of the real-world original work. It should be noted that the sample size of *N* = 38 was very low resulting in low statistical power and thus being unlikely to replicate the result even if there was a true effect and that other factors differing between a real-world interaction with a physical robot or a simulated robot within a VR environment could account for differences in study results. Alternative approaches such as replication studies in VR are far from ideal and have a number of limitations (i.e., it is still a subject of current research to what extent the difference between simulated robots in a VR environment and real interactions with real robots itself produces differences in study results and is therefore problematic, e.g., Roberts et al., 2019; Mara et al., 2021b; Mara et al., 2021a). However, the study shows that there are at least some attempts of replication in HRI user research.

In summary, three replication studies presented here did not replicate the original findings successfully. While every study has its own problems, and a lack of evidence does not mean that the effect does not exist, it suggests difficulties of replications in HRI. They indicate that HRI research might have similar problems as other disciplines. While the direct replication of Ullman et al. (2021) might indicate problems of the robustness of research results, conceptual replications that used other methods to test the same hypotheses as original work can indicate problems in generalizability of results across certain parameters (Leichtmann and Nitsch, 2020b; Wijnen et al., 2020; Ullman et al., 2021). Replications additionally point out problems in the original work that may have led to the failure of replication (e.g., low power of original work) (Leichtmann and Nitsch, 2020b; Ullman et al., 2021). However, the three-site replication study by Strait et al. (2020) is a first example from recent years that alters the question from “if” to “how” HRI user studies are replicable by suggesting processes of sample size justification, cooperation across different labs, and open materials, code and data (Strait et al., 2020).

### 3.4 Some Unique Aspects of HRI Research

In order to estimate the replicability of HRI user studies, we used three different approaches, 1) the structural comparison of publication processes as well as research practices in HRI with processes and practices of those disciplines that already face a serious replicability crisis, 2) the analysis of meta-analyses about empirical HRI studies that reveal parameters and their values that are known to affect replicability, and 3) the description of replication studies of HRI research directly. This analysis showed that HRI has the same problematic research processes and practices that cause replication problems and generally led to a confidence crisis in other disciplines. Especially the analysis of several HRI meta-analyses with different scopes and covering a large amount of studies give strong hints toward this conclusion.

We would also like to emphasize that HRI as a research field has its own characteristics that are related to replicability. Unlike other disciplines, HRI faces the challenge that robots vary across studies, are unique prototypes, are often expensive and complicated to use. Therefore, it is even more difficult to replicate HRI studies with the same robots. Although it is possible to use similar or virtual robots for replication, it is yet unclear how these changes affect replicability (Wijnen et al., 2020; Ullman et al., 2021). Another aspect that distinguishes HRI research from disciplines like psychology is its interdisciplinarity (Sabanovic et al., 2017). While there are some drawbacks of interdisciplinarity, such as a lack of common standards, or a variance in knowledge about statistics, there is also much richness that can help HRI research in tackling problems of confidence. This richness lies in analyses from different perspectives, a rich variety of different methods from different disciplines, or its openness to different scientific approaches that might enable the discipline to rapid change. Finally, especially robotics is a fast growing research field and continuous technological development with potentially transformative character of such technologies (for the transformative character of technology see for example Dolata, 2013 or Hughes, 1987). It might thus be conceivable that research results from past laboratory studies, in which participants had interacted with robots for the very first time in their life and only know robots from science-fiction movies, might not be replicable in future studies simply due to socio-historical changes as robots get more common at work or in our social life. As Gergen (1973) would put it, such results might not transcendent their socio-historical boundaries.

## 4 Toward More Reliable HRI Research

Now that we know that there are replicability problems and problems in confidence in HRI research results when it comes to user studies–what can be done? In the following, we list a row of reforms that have been proposed by scholars from other disciplines and will improve HRI user studies (e.g., Munafò et al., 2017). Note that this article focuses on the part of HRI that aims to understand and predict human behavior, cognition and emotion in context of interaction situations with robots and thus the following reform suggestions are also mainly meant to improve this sort of research in HRI.

### 4.1 Statistical Reforms

A major cause of replicability problems is the misuse and misunderstanding of NHST. For quantitative studies, reforms on the use of statistics are therefore recommended.

#### 4.1.1 Statistical Rigor and the New Statistics

To avoid false-positives, many reformers advocate for more statistical rigor (Wiggins and Christopherson, 2019). That includes to control for multiple testing more consequently in order not to increase the family-wise error rate. Additionally, sample sizes had been found to be low leading to underpowered studies. Therefore, sample sizes in HRI user studies should be justified by a priori power analyses and expected effect sizes need to be reported (Maxwell, 2004; Schimmack, 2012; Button et al., 2013). Furthermore, statisticians call for a stricter alpha level such as *α* < 0.005 (instead of a *α* < 0.05) (Benjamin et al., 2018), or at least to justify the alpha level (Lakens et al., 2018). While higher sample sizes can be challenging especially for HRI research because studies with robots are expensive and difficult to conduct, more cooperation across different laboratories should be fostered such as in the Many Labs Projects (Stower et al., 2021). As one of the first attempts of such a cooperative multi-site replication project in the field of HRI, the study by Strait et al. (2020) was discussed in the previous section. Besides problematization of *p*-values it was argued by reformers that researchers should put more emphasis on effect sizes as they indicate importance of effects as well as confidence intervals indicating uncertainties (Maxwell, 2004; Lakens, 2013; Lakens and Evers, 2014). Other researchers recommend Bayesian statistics as an alternative (Romero, 2019), also in human-machine interaction research (Körber et al., 2016).

#### 4.1.2 Avoid Questionable Research Practices

Another aspect closely related to statistical rigor is to avoid QRPs like p-hacking as these lead to an overestimation of effect sizes and higher false-positive rates. Methods to avoid QRPs include the preregistration of studies, transparency, as well as open science practices that are further explained below (Wiggins and Christopherson, 2019; Scheel et al., 2021a).

### 4.2 Methodological Reforms

#### 4.2.1 Preregistration

Reformers recommend the use of preregistrations of studies (in case the study aims to test a priori hypotheses) to avoid QRPs. For a preregistration, researchers upload a description of study details to a public repository prior to data collection (Scheel et al., 2021a). These details include, for example, the methods they plan to use, hypotheses, planned sample size, or statistical analyses. Consequently, preregistration limits a researcher’s degree of freedom and thus helps to avoid QRPs. Additionally, preregistration fosters transparency of the research process, another important value in improving replicability (Wiggins and Christopherson, 2019). While there have not been many preregistrations of HRI studies in total (Leichtmann and Nitsch, 2020a), the Open Science Framework (OSF) shows that the number of preregistered HRI studies has been increasing over the last 3 years.

#### 4.2.2 Communication: Transparency and Open Science

Transparency is a core value in science that also plays a key role in improving replicability and confidence (Romero, 2019; Wiggins and Christopherson, 2019). Transparency means to disclose detailed information on the research process, disclose all statistical analyses exhaustively, and report all necessary study details. Being transparent also means adhering to the standards of scientific reporting (Romero, 2019). Another consequence of this call for transparency is the pursuit of open science that does not only mean to describe the research process in a report, but even make original materials, the code for data analysis, and data themselves publicly available (Maxwell, 2004; Button et al., 2013). This helps to overcome the confidence problems in multiple ways: Transparency enables researchers to evaluate the quality of research, improves replicability of studies, improves integration of different study results in meta-analyses and helps researchers to better contextualize findings (Wiggins and Christopherson, 2019).

#### 4.2.3 Solid Theory and Validation

Another major driver of the replicability crisis is the lack of theory. Researchers must put stronger emphasis on the development of theoretical frameworks rather than formulating hypotheses based on guesswork (Muthukrishna and Henrich, 2019). Testing hypotheses is only informative when based on theory, but we seem to lack crucial knowledge about auxiliary premises, boundary conditions, causal relationships, measures, and concepts. Scheel et al. (2021b) even think that behavioral scientists may not be ready to test hypotheses yet. Instead, researchers should strengthen the “derivation chain” first. To do this, Scheel et al. (2021b) recommend to put more emphasis on descriptive and naturalistic observation (e.g., to develop a typology), a priori evaluations of theory plausibility (e.g., assess whether a theory is consistent with principles of established theories), parameter range explorations, exploratory experimentation, and conducting pilot studies (Scheel et al., 2021b). Especially qualitative and analytical work is valuable in this respect. Another important step is establishing valid measurement instruments by rigorous validation processes (e.g., Carpinella et al., 2017). These activities are important elements in the “derivation chain”, are valuable and should not just be treated as a necessity for hypothesis testing (Scheel et al., 2021b).

#### 4.2.4 Beyond the Lab: Pluralism of Methods

Wiggins and Christopherson (2019) state that reforms “*have been revolutionary in challenging the norms and practices of psychological science, but have perhaps been less revolutionary in terms of their philosophy of science*”. Many researchers have criticized objectivist epistemology and universalist ontology. Laboratory studies had been found to be biased and error-prone (Orne, 1962; Rosenthal, 1966; Reis, 2019) and are often argued to be of limited validity and generalizability (Greenwood, 1982) because they represent special social contexts that are not representative for natural settings (Eagly and Riger, 2014). Laboratory experiments could thus be viewed as special use cases (Wiggins and Christopherson, 2019).

HRI research could benefit from methods based on philosophical positions other than the current mainstream (Wiggins and Christopherson, 2019). One example is feminist epistemology, which emphasizes that science is shaped by social and cultural contexts and points out the pervasiveness of androcentric biases in scientific practices and communication (Eagly and Riger, 2014). As such, feminist epistemology rejects the notion of an “objective” science, but challenges the values, norms and assumptions that inform our research by critical reflections and questioning the research questions, questioning the language we use in the scientific processes, or by locating the researcher in the research process (Wigginton and Lafrance, 2019). In HRI, too, there are already first articles grounded in feminist epistemology that could be further explored in future work (Weber, 2005; Winkle et al., 2021). Another example is critical psychology that also recognizes that theories, methods, and practices are determined by socio-historical contexts. Critical psychology, thus, proclaims problem-centered research with an activist dimension to address real-world social problems (Teo, 2015). Similarly, we as HRI researchers can also pursue a more activist dimension by using robot design as well as interaction design in order to challenge current social problems, to foster certain human values, or to raise awareness for certain issues for instance. Especially for HRI, design epistemology will lead to valuable insights. HRI is an applied research field that is focused on the design of real-world interactions between humans and robots meeting certain values. HRI research could also emphasize its active component more strongly. For example, knowledge (on human behavior in HRI contexts) can also be constructed by actively designing human-centered HRI (an approach also known as “research through design”, see Zimmerman et al., 2007), while accompanying these activities with critical reflections and documentation (Lupetti et al., 2021) or participatory design (Rogers et al., 2021). For example in work by Gollob et al. (2021), the researchers explored new aesthetic experiences by creating new artefact interactions in different projects. Such “designerly ways of knowing” had been overlooked in HRI research for some time as HRI research often focused on evaluative studies using quantitative methods (Lupetti et al., 2021). However, design epistemology and design methodologies can improve knowledge in HRI in form of tools, guidelines, criticisms, concepts or annotated portfolios (Lupetti et al., 2021).

HRI research aiming to explore human behavior, cognition or emotion in interaction situations with robots should hence more strongly value other research activities beyond hypothetico-deductivist approaches. This includes methods other than quantitative evaluation studies such as qualitative, analytical, or theoretical work (e.g., Veling and McGinn, 2021). Additionally, HRI research in this context should also focus more on holistic research programs that examine an interaction context in-depth covering field and longitudinal studies. Finally, HRI as a community currently also widely lacks systematic analysis of the practices used in HRI, researchers’ scientific understanding or the analysis of the HRI community as a social system. As science is itself a social system metascience (a growing field of research) is needed that documents and analyses the methods and practices that are used in the HRI community, how research is communicated, or how it is verified, evaluated and incentivized and then gives corrective recommendations for improvement (Ioannidis et al., 2015). Metascience is thus not to be confused with meta-analysis that summarizes effects in the literature on a certain research question quantitatively, but metascience means the science about the science and “*involves taking a bird’s eye view of science*” (Ioannidis et al., 2015). The way we as HRI researchers conduct, report, verify, evaluate and incentivize research needs to be documented and analyzed in order to be corrected if needed (see Elson et al., 2020 as an example of a metascientific experiment).

#### 4.2.5 Robustness Checks

While research is often focused on novelty, the replicability crisis shows that focus on the assessment of robustness is necessary (Open Science Collaboration, 2015). Therefore, more effort is needed to conduct replication studies to investigate the stability of HRI findings and to explore if an effect holds under different conditions (e.g., different cultures). This can be achieved by large scale replication attempts with multiple laboratories following the model of the Many Labs Projects (Munafò et al., 2017; Klein et al., 2018). Robustness checks do not have to be replication studies. Other ways to test the robustness of results are checking statistical results for inconsistencies, re-analysis of results by other researchers, or sensitivity analysis (Nuijten, 2021).

### 4.3 Social Reforms

It was emphasized that science is a social system, and that the replicability crisis also has social causes requiring social reforms. For example, problems in publication processes and incentive systems cause publication bias.

#### 4.3.1 Establishing Standards

One way to tackle publication bias through social changes is to establish other incentive systems. Instead of novelty or the outcome of a study, articles submitted for publication should be evaluated on the quality of the work. However, especially in interdisciplinary research fields, there are not many common standards. Nevertheless, the HRI community is becoming aware of the need to improve the quality of methods, statistical analyses, as well as completeness of reporting. Thus, scholars have started to develop standards and guidelines, such as guidelines in reporting statistics (Hoffman and Zhao, 2021), or in reporting the context of human-robot interaction (Onnasch and Roesler, 2021).

#### 4.3.2 Change in Incentives and Publication Processes

Instead of rewarding work based only on its novelty value or outcome, work that tests the robustness of results (such as replication studies) or measures that ensure greater reliability of studies such as open science practices, preregistration, or validation efforts should also be encouraged. This requires increasing awareness of funding agencies and scientific journal editors (i.e., that do not reject work because of results being non-significant, or using non-quantitative methods). The HRI community is well on its way by valuing replication studies and opening up to other epistemological and methodological approaches beyond laboratory and questionnaire studies. For example, the *ACM/IEEE International Conference on Human-Robot Interaction* in 2020 recognized issues of reproducibility as one of the main themes. Additionally, the HRI research community also deliberately valued a diversity in research approaches in the past by explicitly highlighting the interdisciplinary nature of the field. Interdisciplinary research collaborations can foster reforms needed to tackle a potential crisis of confidence. This requires epistemic and methodological openness and understanding, which could be achieved through interdisciplinary training and the necessary funding and infrastructure for such larger projects with researchers from multiple disciplines.

### 4.4 Where does HRI Stand and What Needs to be Done

It needs to be mentioned that the crisis of confidence and related reforms mostly concern hypothetico-deductivist research such as NHST. Analyses show these methods are also common in HRI (Baxter et al., 2016) and first evidence presented in this article indicates that HRI may likely face similar problems. This does not mean that HRI researchers should not test hypotheses, but that more reflection, rigor, and openness to alternative approaches is necessary. HRI is a young, interdisciplinary field with the opportunity to tackle problems early. Indeed, the HRI community is already taking steps toward greater reliability in multiple ways, including workshops, critical reflections by scholars, or a growing interest in alternative methods. For example, Eyssel (2017), Belpaeme (2020) or Hoffman and Zhao (2021) point out problems of current HRI research practices and highlight the need for statistical rigor and for theory development. Similarly, most of the authors of the meta-analyses reported in this article also discussed problems of the original studies and gave several recommendations for future research in their discussion sections (e.g., Leichtmann and Nitsch, 2020a; Mara et al., 2021; Stower et al., 2021). Additionally, a growing number of conference workshops on test methods and metrics (Marvel et al., 2020; Marvel et al., 2021), on design research (Luria et al., 2021) or ethnography (Hasse et al., 2018) sought to develop new metrics and explored unconventional methodology—endeavors that are also echoed in HRI articles on methods (Alves-Oliveira et al., 2021; Lupetti et al., 2021; Rogers et al., 2021; Veling and McGinn, 2021) or standards (Fischer, 2021; Hoffman and Zhao, 2021; Seibt et al., 2021). Furthermore, especially the *ACM/IEEE International Conference on Human-Robot Interaction* emphasized the importance of replication by introducing a theme on “Reproducibility of Human-Robot Interaction” in 2020 and by explicitly calling for submissions of work focusing on replicability of HRI studies in the last few years. Many of these discussions and reform steps, such as a growing number of replication studies being conducted (note that most replication studies reported in this article had been published between 2020 and 2021) or multisite projects (e.g., Strait et al., 2020), as well as a growing number of preregistrations of HRI studies, have been more common especially in recent years showing that HRI research is already in change. However, in particular reforms such as requiring higher sample sizes take more effort and cause higher costs for study designs and can for this reason also have negative side effects if, for example, the number of low-cost online studies (without real interaction) increases in order to meet high standards of sample size (a development that has been observed in social psychology in the past years, Sassenberg and Ditrich, 2019) and studies with actual interactions between robots and humans decrease due to the difficulties and costs. HRI must therefore be careful not to lose the inner essence of the real-world interaction between humans and robots, its design mission, and the essence of its openness to methods, approaches, and perspectives, while still maintaining appropriate standards to improve the quality of research results as reported in the previous section on reforms.

Based on our analysis from the perspective of the behavioral sciences, we would summarize major learnings as follows: More general, the social system of HRI as a discipline needs some changes. Collaborative and interdisciplinary projects, open science, and replication studies should be incentivized. The publication system should allow for a greater variety of methodological approaches and at the same time value common standards (e.g., statistical rigor). To foster this, standards for interdisciplinary HRI research and respective trainings for HRI researchers need to be developed. Furthermore, HRI would benefit from more openness toward work that stands apart from the current mainstream, such as research grounded in alternative epistemologies (e.g., feminist epistemology), methods allowing for insights from other disciplines (e.g., design research), and methods that support theory development, including analytic, theoretical, and reflective work, as well as qualitative and exploratory research. Hypothetico-deductive research and lab experiments remain important within HRI. However, when applying these methods, solid theoretical foundation of hypotheses and statistical rigor (e.g., a priori power analysis) need to be considered. If this is not possible, chances are that the field is not yet ready to test hypotheses and should rather work on other (before mentioned) aspects (Scheel et al., 2021b). Regardless of the chosen method, researchers should put more emphasis on transparency, open science (e.g., open materials/data), replication and robustness checks. The development of the research field will also benefit from metascientific monitoring that is its methods, research communication, as well as its verification, evaluation and incentivization processes need to be documented, analyzed and critically reflected.

## 5 Conclusion

HRI covers a rich body of research efforts from various perspectives and disciplines. In order to evaluate the interaction between human users and new robots quantitative user studies are widespread in the community. This article showed that HRI user studies likely face replicability problems of its own similar to other disciplines such as the behavioral sciences. Although replication studies are rare in HRI, first examples showed difficulties in replicating results from original work. More reliable evidence from meta-analyses showed that HRI studies share the same problematic structures and practices that contributed to the replicability issues in other fields. This raises doubts on the stability and generalizability of results. It is therefore important that HRI as scientific discipline increases efforts to take corrective action. The HRI community is already on a good way: The openness to different epistemological and methodological approaches and the richness of perspectives through its interdisciplinarity can have a promising effect on dealing with such problems of confidence in study results. Nevertheless, reforms are needed, for example, in the form of quality checks or the development of better theoretical foundations. Even small and easy changes such as sharing the analysis code can be effective.
